# Importance and Diagnosis of Flexibility Preparation of Male Sport Climbers

**DOI:** 10.3390/ijerph17072512

**Published:** 2020-04-07

**Authors:** Paweł Draga, Mariusz Ozimek, Marcin Krawczyk, Robert Rokowski, Marcelina Nowakowska, Paweł Ochwat, Adam Jurczak, Arkadiusz Stanula

**Affiliations:** 1 Kletterverband Österreich, 6020 Innsbruck, Austria; paweldraga@wp.pl; 2 Institute of Sport, Department of Track and Field’s Sports, University of Physical Education, 31-571 Krakow, Poland; mariusz.ozimek@awf.krakow.pl; 3 Faculty of Health Sciences, University of Applied Sciences, 33-100 Tarnow, Poland; m_krawczyk@pwsztar.edu.pl; 4 Department of Tourism and Leisure, Section of Mountaineering and Qualified Tourism, University of Physical Education, 31-571 Krakow, Poland; robert.rokowski@awf.krakow.pl; 5 Institute of Sport—National Research Institute, 01-982 Warsaw, Poland; marcelina.nowakowska@sport-olimpijski.pl; 6 Department of Theory and Methodology of Physical Education, University of Physical Education, 31-571 Krakow, Poland; pawel.ochwat@awf.krakow.pl (P.O.); adam.jurczak@awf.krakow.pl (A.J.); 7 Institute of Sport Science, Department of Exercise and Sport Performance, The Jerzy Kukuczka Academy of Physical Education, 40-065 Katowice, Poland

**Keywords:** sport climbing, redpoint, flexibility, climbing-specific fitness tests

## Abstract

The objective of the study was to verify the relationships between sport skill levels and to identify the tests that accurately diagnose flexibility of sport climbers. This study examined 60 competitive advanced–higher elite male 7b–9a redpoint (RP) climbers. The athletes performed commonly used flexibility tests (stand-and-reach, straddle sit, straddle stand) and climbing-specific flexibility tests. Significant correlations were found between sport skill levels for the straddle stand test (r = −0.48) and the straddle sit test (r = −0.41). No significant correlations were observed between climbing-specific flexibility tests and sports skill level of climbers. Hip abduction evaluated using the straddle sit and straddle stand tests were significantly correlated with sports skill level and thus can be approached as a tool to diagnose flexibility of climbers. Flexibility is very specific and difficult to diagnose in climbing, but it should be developed.

## 1. Introduction

Climbing has developed as both a competitive sport and a recreational physical activity [1,2]. The International Olympic Committee (IOC) and the International Federation of Sport Climbing (IFSC) announced that climbing would become an Olympic sport in 2020 [3]. Improved safety and modern training equipment have led to substantial progression in the level of difficulty of new climbing routes. Dynamic development of climbing as a sport and activity, as well as the inclusion of the sport into the Olympic program, encourages research on the factors that determine the achievements of the best climbers.

Sport climbing is considered an endurance and strength sport with complex movement biomechanics [4,5]. The significant effect of fitness-related abilities on the level of achievement of climbers has been emphasized by numerous researchers [1,6,7,8]. The major determinants of success in climbing include finger and arm strength, muscle endurance [1,7,9], specific hip mobility [10], and characteristics of body build, such as low body mass, low body fat, and average body height [9,11,12]. Flexibility has been identified as one of four abilities that determine success in climbing [13,14,15,16,17,18,19]. There are six kinds of flexibility, with classification depending on the character of the muscle action and on the presence and absence of an external force that aids moving throughout the range of motion a stretched position [20]. The six kinds are: (1) dynamic active flexibility, (2) dynamic passive flexibility below the pain, (3) dynamic passive flexibility over the pain threshold and up to pain tolerance, (4) static active flexibility, (5) static passive flexibility below the pain, and (6) static passive flexibility over the pain threshold and up to pain tolerance. Hip joint mobility is expected to play a particular role. Goniometric measurements of the hip joints of male climbers were performed by Grant et al. [19] and in females by Mermier et al. [14], who obtained various results suggesting that the ranges of hip mobility can explain sport results obtained by climbers only to an insignificant degree. Furthermore, Grant et al. [13] used a climbing-specific test (Grant foot raise) but did not find significant differences between elite and recreational female climbers. Attempts to verify Grant’s sport-specific test [13] were made among male and female climbers by Draper et al. [21], who failed to establish a statistical significance with competitive performance (r = 0.20, *p* > 0.05), which led the authors to develop new tests. The tests designed by these researchers included the adapted version of Grant’s test [19], climbing-specific foot raise, lateral foot reach, and foot loading tests. The authors obtained significant correlations in the foot rise test and foot loading test (r = 0.53, r = 0.56; *p* < 0.05). Analysis was also performed for the correlations of the standard tests with those designed specifically for climbing. Standard tests were found unsuitable for the measurement of climbing-specific flexibility while their diagnostic value is yet to be verified. The results of the above mentioned research indicate that the issue of the importance of flexibility in sport climbing still requires research. The latest research, carried out on a large sample (44 men and 33 women), by the MacKenzie et al. [22], indicates gender-dependent gradation of the flexibility compounds (measured with non-specific and specific tests) with a change in sport climbing levels. Significant correlations were noted in the male group. MacKenzie et al. [22] concluded from the results of their research that flexibility belongs to the group of secondary determinants. 

The studies by Grant et al. [13,19] and Draper et al. [21] were based on a relatively small sample of climbers (respectively: 10 elite climbers, 10 recreational climbers, and 10 non-training athletes and 5 elite competitors). As it can be seen, the latest results of the research on the importance of flexibility, not counting the work of MacKenzie et al. [22], in which they studied more climbers, were published by Mermier et al. [14]. In light of the analysis of available literature, there are no studies with a wide range of measurements and a large number of participants in which correlations of the level of flexibility with the level of climbing advancement were assessed.

Therefore, detailed analyses in this respect may be available and provide (1) high cognitive value enriching knowledge of climbing sports; (2) significant importance in coaching practice, in the selection of the most informative tests; and (3) enable control of the dynamics of the development of flexibility in climbers at different levels of advancement. The main aim of the study was to determine the correlations between the sport skill level and the flexibility of climbers. The secondary aim was to verify the diagnostic values of selected sport-specific and general tests of flexibility for advanced and elite climbers.

## 2. Materials and Methods

**Figure 1:**
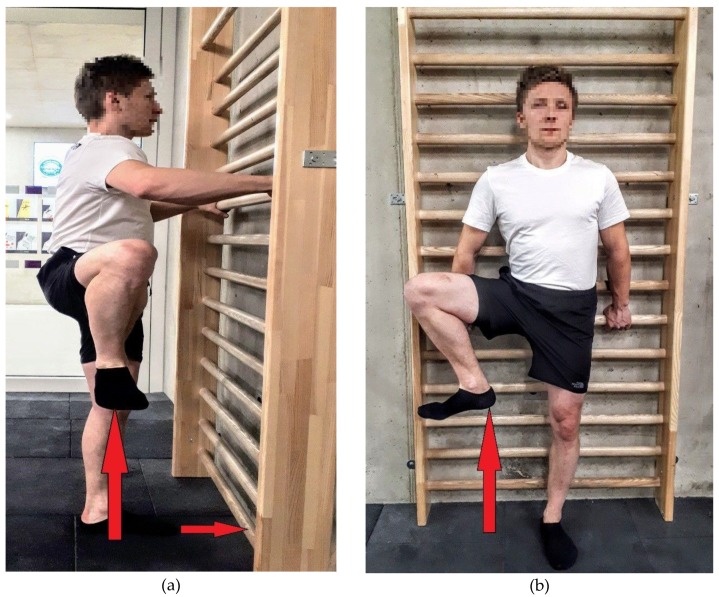
Tests for evaluation of hip joint mobility: (**a**) Grant test modified by Draper et al. (2009); (**b**) Author’s Draga test.

The study examined two selected groups of male climbers. In the first group of 7b–8c redpoint (RP) climbers (*n* = 29), we used classic flexibility tests, such as the straddle stand test, straddle sit test, and the stand-and reach test. In the second group, characterized by the 7b–9a RP sports skill level (*n* = 31), we used the Grant test (Figure 1a) and the author’s test and its index (Figure 1b), and measured the length of the lower limb as a distance from base to trochanterion in cm (B-tro). The selection of different test sets for both groups of climbers was aimed: (1) to verify the diagnostic value of classical flexibility tests on a large group of climbers with a high sporting level, and (2) to verify, on a large sample, specific tests dedicated to assessing flexibility at the elite climbing level. The age of study participants ranged from 20 to 39 years, whereas their competitive experience was at least 3 years. A warm-up was performed individually after previous recommendations of specific and general flexibility exercises. After ethics approval by the Krakow Medica Ethics Committee (42/KBL/OIL/2015) in accordance with the Helsinki Declaration, the experimental protocols were explained and consent was obtained from each participant.

### 2.1. Description of the Tests in the Group of 7b–8c RP Climbers

1. Maximal straddle—evaluation of hip joint mobility (cm). The participant performed a maximal straddle in the frontal plane, with feet flat on the ground and the legs kept straight. The shortest distance from the pubic symphysis to the ground was measured.
2. Straddle-sit—evaluation of hip joint mobility (cm). The participant performed maximal straddle-sit with his face facing the wall, feet rested on the wall and knees straight. The shortest distance from the pubic symphysis to the wall was measured.
3. Stand-and-reach—evaluation of the sacroiliac joint mobility, spinal mobility, and flexibility of the hamstrings (cm). The participant performed a maximal stand-and-reach exercise while standing on a platform. The shortest distance from the longest finger of the palm of the hand to the ground was measured.

### 2.2. Description of the Tests and Measurements of Somatic Characteristics in the Group of 7b–9a RP Climbers

1. Lower limb length (B-tro)—length from base to trochanterion point in cm.
2. Grant test modified by Draper et al. [21] (Figure 1a)—evaluation of hip joint mobility (cm). The study participant stood at the distance of 23 cm from the wall with his palms resting on the wall with the fingers up. The participant performed lateral rotation of the foot with his toes up and lifted the lower limb bent in the knee joint as high as possible. The distance from the uppermost foot point to the ground was measured.
3. Author’s “Draga” tests (Figure 1b)—evaluation of hip joint mobility (cm). The back of the study participant was touching the measurement board hung on gymnastics wall bars, with his pelvis stabilized with a waist belt attached to the board. The arms of the participant were along the sides of the body and hands held a wall bar, which also stabilized the body trunk. Feet were positioned perpendicularly to the table, with the heels touching the 3 cm limiter attached to the bottom of the board. The participant performed rotation of the right foot to the outside and a maximal raise of the leg bent in the knee. The distance was measured between the ground and the heel (section a). The results were recorded in absolute and relative values using the index of the rise of the lower limb termed the Draga-index (DI), given by the following formula:
  $$DI=\frac{B-tro}{a}$$
  where: B-tro—lower limb length (cm); a—length of the section between the calcaneal tuberosity and the ground (cm).

### 2.3. Statistical Analysis

Measurement data was subjected to statistical calculations. Arithmetic means (m), standard deviations (SD), minimal and maximal (min–max) values, and Pearson’s linear correlation coefficients (r) were calculated. The Pearson’s correlation was justified since the level of sport skills in climbing corresponds to the level of route difficulty. We used the French scale, which does not contain the International System of Units (SI) but only conventional ordinal values. The conversion factor was used to convert the French grades into the Watts point scale [23]. We also employed the taxonomic analysis to determine the validity of flexibility tests for climbers. All statistical computations were performed by means of the STATISTICA software ver. 9.0 (StatSoft^®^, Tulsa, OK, USA).

## 3. Results

Descriptive statistics of the classic climbers’ test 7b–8c RP for the first group of climbers (*n* = 29) are presented in Table 1. According to the presented data the competitors achieved average results of 21.92 ± 12.08 cm in the stand-and-reach test, 38.15 ± 12.27 cm in the straddle-stand test, and 43.79 ± 11.4 cm in the straddle-sit test.

The resulting descriptive statistics of sport skill levels, lower limb length, and sport-specific flexibility tests are presented in Table 2. In the second group, characterized by the 7b–9a RP sport skill level (*n* = 31), the RP level had a mean score 3.91 ± 0.74, the lower limb length test a mean of 91.47 ± 4.29 (cm), the Grant test a mean of 97.25 ± 8.79 (cm), the Draga test a mean of 74.07 ± 6.26 (cm), and Draga-index a score of (DI) 0.81 ± 0.07.

The results obtained using general flexibility tests were significantly correlated with the sport skill level of climbers (Table 3). The most significant correlations with sport skill level were found for the maximal straddle-stand test (r = −0.48), whereas the least significant was the maximal straddle-sit test (r = −0.41).

**Figure 2:**
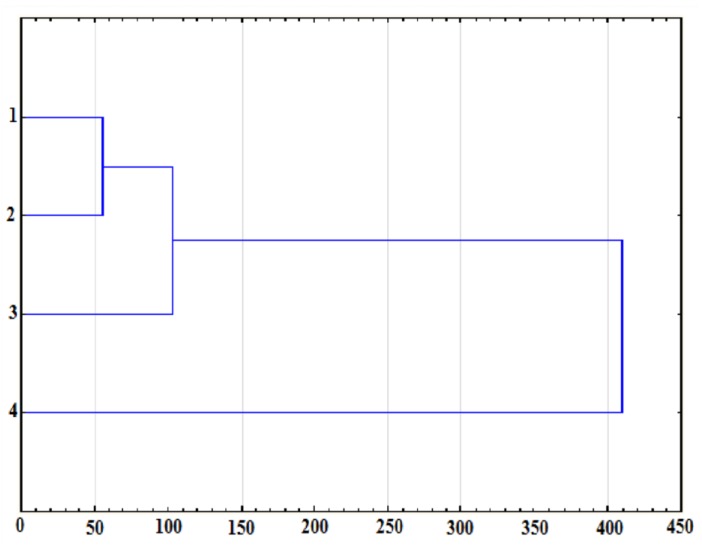
Ward tree diagram for 7b–9a RP climbers: (1) B-tro, (2) flexibility test (Grant), (3) author’s flexibility test (Draga test), (4) Draga-index (DI).

Taxonomic analysis of climbing-specific tests with consideration for the somatic variable (lower limb length) was also performed. A relationship was found in the dendrogram (Figure 2) between the Grant test (raise of the foot rotated outward) and the lower limb length. The author’s Draga test (raise of the rotated foot with stabilized pelvis) demonstrated a relationship to the Grant test. The relative value of the author’s test (DI) did not reveal a linkage at a low level with other tests or with the length of the lower limb.

The sport-specific tests supported the results of the taxonomic analysis concerning the correlations of the Grant test with lower limb length (r = 0.35) and Draga-index (r = 0.63). No significant correlations were found between sport-specific test results and sports skill level (Table 4).

## 4. Discussion

The use of flexibility training in order to prevent injuries [24,25,26,27] and to improve sports performance is very common [21,28,29]. From sports in which aesthetics determine the score (dancing, rhythmic gymnastics, artistic gymnastics) to those oriented around efficiency of activity (combat sports, running), substantial range of motion in the hip joint is conducive to success in competition. In addition to the positive effect on sports performance, the wide range of motion can also represent an adverse or even pathological phenomenon and lead to hypermobility and, consequently, damage to the joint. Pathological changes caused by flexibility training have been mostly observed in sports where training has to be started early and where this ability is one of the major determinants of success [28,30]. Contemporary sport climbing is demanding with respect to the abilities of the lower limbs, starting from the hip joint through to the feet phalanges. A plethora of climbing techniques requires raising the lower limb high, substantial rotation, maximal abduction, and maintaining the athlete’s body in extreme positions. Measurements of hip joint mobility and its effect on the level of competitive performance in climbers have been extensively explored by such researchers as Grant et al. [13,19], Watts [15], Sheel [16], and Giles et al. [18] as a highly significant factor. Most authors approached flexibility as one of the determinants of competitive success in climbing. However, its role as a separate factor failed to be examined. A more in-depth analysis of flexibility as a separate factor was made by Draper et al. [21]. The small number of publications encourages further scientific research concerning flexibility of sport climbers.

A significant effect on sports skill level of climbers was also observed in the present study. The hypothesis that sport-specific flexibility tests (the modified Grant test, the Draga-index test) would be substantially correlated with the sport skill levels of climbers was not supported. The results of the studies should be compared to the results obtained by Draper et al. [21]. These authors used the modified Grant test and obtained an insignificant correlation with sport skill level (r = 0.31). In our study, both the Grant test and the author’s test with the structure similar to the Grant test (the Draga test) were not significantly correlated with sport skill levels of climbers. The lack of significant correlations of special tests should be further analyzed.

The non-specific stand-and-reach test, which has been commonly used in various areas of human physical activity, was significantly correlated with sports skill level of climbers (r = 0.46). The stand-and-reach test was previously used by Grant et al.: *p* = 0.23 [13] and *p* ≤ 0.05 [19], Mermier et al.: *p* ≤ 0.05 [14], Draper et al.: *p* ≤ 0.05 [21], and Espana-Romero et al.: *p* ≤ 0.001 [31] and was not significantly correlated with sport skill levels of climbers. In the study by Espana-Romero et al. [31], the researchers demonstrated a substantial difference in the results of this test between groups of 7b–8b male climbers and 7a–8a female climbers. Draper et al. [21] found that with the increase in sport skill level, the results obtained in the stand-and-reach test were improved, whereas correlations with sport skill level became weaker (r = 0.15, ns.). Significant differences in sport skill levels between climbers are likely to be reflected by general fitness, which determines special fitness. Sport skill level was also a distinguishing factor for the group which participated in the study of advanced to higher elite International Rock Climbing Research Association (IRCRA) climbers [32]. Correlation between stand-and-reach test results and competitive performance was r = 0.46 (*p* ≤ 0.05). However, these findings are inconsistent with the previous results, and other results are probably due to large differences in the levels of climbers tested and not considering the gender. Draper et al. [21] referred to the studies by Jackson and Baker [33] and Jackson and Langford [34], which demonstrated that the stand-and-reach test evaluates flexibility of the hamstrings rather than the hip joint mobility that is important in sport climbing. This author found that the test is not suitable for the measurement of climbers’ flexibility despite recommendations for other sports, including team sports [35].

The results obtained for the stand-and-reach test were caused by several factors, such as anthropometric dimensions [36], test protocols [37], methodological variables [38], mobility of spinal segments, and angle of pelvic inclination [39,40]. Numerous factors are likely to modify the results of the stand-and-reach test, and this provokes the next questions for future research.

Hip abduction tests (straddle stand and straddle sit) reached statistical significance with sports skill level. The results seem to be justified by the type of techniques used by climbers. Climbing routes during competition and in natural terrain often require positioning the athlete’s legs on footholds across substantial distances. The substantial distance between footholds engages not only passive flexibility, when the athlete adopts the resting position, but also active flexibility, when the athlete performs broad leg movements in order to move on the climbing route.

The specific test of maximal lateral foot reach was performed by Draper et al. [21] using the climbaflex system. This was probably the first sport-specific test for lateral foot reach in the climbing situation. The authors obtained a significant correlation of this test with competitive performance of climbers, but only relative to body height.

## 5. Conclusions

The results obtained in the stand-and-reach test are not statistically correlated with the athletes’ sport skill levels. This may be due to the lack of consideration of the length factor of the upper body relative to the lower, which should be included in further research. Hip abduction evaluated using the straddle sit and straddle stand tests were significantly correlated with sport skill level in male climbers (7b–8c redpoint) and thus can be approached as a tool to diagnose flexibility of climbers. However, it should be noted that the majority of the correlation coefficient is obtained in the straddle-stand test. Such a test is more similar to the discipline requirements than the straddle-sit test. Specific Grant tests, contrary to the results presented by other researchers, did not achieve significant correlations with the sport level of climbers [21]. In this study we have shown that the originally proposed Draga test and its index sport level of investigated climbers is not statistically significant. Lack of significant correlations of specific tests prevents their use in practice but can be a motivation for their subsequent verification and modification.

## Figures and Tables

**Table 1:** Descriptive statistics of classic tests for 7b–8c redpoint (RP) climbers (*n* = 29).

Variable	m ± SD	Min–Max
Stand-and-reach (cm)	21.9 ± 12.08	17.5–23.0
Straddle-stand (cm)	38.2 ± 12.27	10.6–53.2
Straddle-sit (cm)	43.8 ± 11.40	12.7–55.4

**Table 2:** Descriptive statistics of sports skill level, lower limb length, and sport-specific flexibility tests for 7b–9a RP climbers (*n* = 31).

Test	m ± SD	Min–Max
RP level	3.9 ± 0.74	3.3–5.5
B-tro (cm)	91.5 ± 4.29	86.0–102.0
Grant (cm)	97.3 ± 8.79	77.0–113.5
Draga (cm)	74.1 ± 6.26	60.0–90.0
Draga-index (DI)	0.81 ± 0.06	0.68–0.95

**Table 3:** Correlations of flexibility tests with sport skill levels for 7b–8c RP climbers.

Variables	Stand-and-Reach	Straddle-Stand	Straddle-Sit
Sport skill level	0.46	−0.48 *	−0.41 *
Stand-and-reach	1	−0.33	−0.25
Straddle-stand	−0.33	1	0.87

* Significant correlations at the level of *p* ≤ 0.05 were highlighted in bold.

**Table 4:** Pearson’s linear correlations of motor variables and lower limb length with sport skill level on the Watts RP scale in the group of 7b–9a RP climbers.

Test	Lower Limb	Grant	Draga	Draga-Index (DI)
Sport skill level	−0.04	0.29	0.17	0.19
B-tro	1	0.35	0.32	−0.21
Grant	0.35	1	0.63 *	0.43
Draga	0.32	0.63 *	1	0.85 *

* Significant correlations at the level of *p* ≤ 0.05 were highlighted in bold.
